# A Multi-Valent Hantavirus Vaccine Based on Recombinant Modified Vaccinia Ankara Reduces Viral Load in a Mouse Infection Model

**DOI:** 10.3390/vaccines13030270

**Published:** 2025-03-04

**Authors:** Marilyn Aram, Victoria Graham, Emma Kennedy, Emma Rayner, Roger Hewson, Stuart Dowall

**Affiliations:** UK Health Security Agency (UKHSA), Porton Down, Salisbury SP4 0JG, UK; marilyn.aram@ukhsa.gov.uk (M.A.); emma.kennedy@ukhsa.gov.uk (E.K.); roger.hewson@ukhsa.gov.uk (R.H.)

**Keywords:** hantavirus, vaccine, efficacy, Seoul virus, Hantaan virus

## Abstract

**Background**: Old World orthohantaviruses are the aetiological agent of Haemorrhagic Fever with Renal Syndrome (HFRS) disease. Worldwide, the two most prominent pathogens of HFRS are Seoul orthohantavirus (SEOV) and Hantaan orthohantavirus (HTNV). There is currently no specific treatment nor widely licensed vaccine form hantaviruses. **Methods**: This study developed a virus-vectored vaccine approach using modified vaccinia Ankara (MVA) incorporating a SEOV-HTNV chimeric nucleoprotein antigen. **Results**: The vaccine demonstrated the induction of humoral and cellular immunity. In the absence of a disease model, a reduction in the viral load of a susceptible mouse strain with type-I interferon receptor deficiency (A129) was used to ascertain protective effects after challenge with SEOV. Results demonstrated a significant reduction in and/or clearance of viral RNA in immunised animals. **Conclusions**: An MVA viral vector vaccine incorporating the nucleoprotein as antigen offers a promising approach for Hantavirus vaccine development.

## 1. Introduction

Orthohantaviruses are a genus of tri-segmented negative-sense, single-stranded enveloped RNA viruses in the *Bunyavirales* order and *Hantaviridae* family that were first recognised as an aetiological agent of viral haemorrhagic fever (VHF) in Europe and Asia, more than 40 years ago [1]. The genome segments termed S (Small), M (Medium) and L (Large) encode the viral nucleoprotein (NP), glycoproteins (Gn and Gc) and an RNA-dependent RNA polymerase, respectively. The S segment also encodes a non-structural protein. Orthohantaviruses are rodent-borne, and their dissemination is mirrored by the geographical distribution of their rodent reservoir. This global distribution has led to hantaviruses being divided into two large groups, namely New World orthohantaviruses, such as Andes and Sin Nombre virus that are found in the Americas, and Old World orthohantaviruses that are predominantly found in Europe and Asia. Sequence-based phylogeny and taxonomy has now formally classified orthohantaviruses into more than 50 species, 20 of which are known to be pathogenic to humans [1].

Clinically, Old World orthohantaviruses are the aetiological agent of Haemorrhagic Fever with Renal Syndrome (HFRS), a clinical syndrome characterised by the sudden onset of headache, fever, nausea, blurred vision and back and abdominal pain, progressing in severe cases to acute kidney failure, vascular leakage and death. The symptoms of HFRS classically appear 7 to 14 days following exposure but can take up to 8 weeks to appear, and the severity of disease varies depending on the aetiological agent. In Europe, Seoul (SEOV), Saaremaa (SAAV), Dobrava (DOBV) and Puumala (PUUV) orthohantaviruses are the most commonly circulating orthohantaviruses. Puumala is the most prevalent and the main etiological agent of Nephropathia Epidemica (NE), a milder form of HFRS (0.5% mortality). SAAV causes a syndrome similar to NE. Dobrava virus (DOBV) causes the most severe form of HFRS in Europe (10% mortality), but human infection is uncommon and appears to be limited to Southeast Europe. SEOV causes HFRS worldwide and is of particular importance in Asia, where orthohantavirus infections are a public health issue; the majority of orthohantavirus cases are in China [2]. The two most prominent aetiological agents of HFRS in humans worldwide are *Hantaan orthohantavirus* (HTNV) and *Seoul orthohantavirus* (SEOV). HTNV has a fatality rate of <15% [3,4], and although SEOV has a lower fatality rate of ~1%, recovery can be lengthy, taking as long as six months with chronic renal failure and requiring dialysis in approximately 20% of patients with SEOV-induced HFRS and in approximately 40% of patients with HTNV-induced HFRS [1].

HTNV, the original species of the orthohantavirus genus, was first isolated in 1978 from an infected striped field mouse (*Apodemus agrarius*) near the Hantaan river in Korea [5,6]. The striped field mouse is the natural reservoir of HTNV [7], which is indigenous to Asia but can also be found across Europe. Meanwhile, SEOV, the primary cause of HFRS in Asia, is vectored by the ubiquitous brown rat (*Rattus norvegicus*) and black rat (*Rattus rattus)*. Following its first isolation in Korea, SEOV has been shown to have a global distribution with clinical cases across Europe and North America. This includes the isolation of viable virus in the UK in 2012 from both wild and pet rats (*Rattus norvegicus domestica*), designated SEOV Humber and SEOV Cherwell, respectively [8,9]. Following this initial discovery of pet rat-associated hantavirus transmission in the UK, further countries, including The Netherlands, Canada and North America, reported similar discoveries. Thus, while SEOV appears to be the most prevalent orthohantavirus infecting rodents in the UK [10], it is also a global zoonotic problem exacerbated by pet rats. The rodent reservoirs of hantaviruses are thought to be chronically, and asymptomatically infected, shedding the virus in their excreta and bodily fluids throughout their life. The virus is maintained in the rodent population by the inhalation of dried, aerosolised infectious excreta and direct contact with infected bodily fluids (e.g., via bites) [8,9,11,12,13,14,15].

Humans are thought to be dead-end hosts for HFRS-associated orthohantaviruses and are primarily infected via the inhalation of aerosolised infectious rodent excreta. Communities at higher risk to hantavirus infection are therefore those that live alongside rodents or those that work in areas inhabited by rodents, for example, sewer workers, military personnel, farmers or foresters [16]. More recently in Europe, owners of the increasingly popular pet, the fancy rat, have also been shown to be at increased risk of hantavirus exposure [17,18]. It has also been observed that climate conditions have had an impact on the incidence of hantavirus infections, where warmer years have resulted in a greater number of infections recorded [19]. In this context, as global warming causes European countries to become more temperate, the need for effective treatments and vaccines becomes more urgent.

There is currently no specific treatment or licensed vaccine for hantaviruses in Europe, the only preventive measure being rodent control. In Korea, there is a formalin-inactivated vaccine against SEOV and HTNV, Hantavax™, available, but this has been shown to have an inconsistent safety profile, where local and systemic adverse events occurred in 47.79% and 25.22% of study subjects, respectively [13]. Furthermore, it provides limited long-term protection against the viruses and is now only recommended for high-risk individuals [20,21].

Recombinant virology techniques have led to the development of fast-track vaccine pipelines suitable for the rapid development and screening of potential vaccine candidates. One such platform/pipeline is the modified vaccinia Ankara (MVA) vaccine vector. Historically, vaccinia virus was used in the original Smallpox vaccine campaign, but there were safety concerns in immunocompromised individuals, and its use as a vaccine was discontinued. In response to this, an attempt to attenuate vaccinia virus by serial passage in tissue culture was undertaken, and, following over 500 passages in chicken embryo fibroblast (CEF) cells, MVA was created. During the passaging process, vaccinia virus underwent a significant loss of genetic information of approximately 15%, and the resultant MVA is unable to replicate in any non-avian cell line. This attenuation of MVA prevented replication in humans [22], and so dramatically improved its safety profile [23]. MVA has subsequently been used to develop vaccines against Influenza, HIV, Lassa and Crimean–Congo Haemorrhagic Fever (CCHF) [24,25,26,27]. In addition, MVA has been shown to stimulate an enhanced IFN-γ response, which is vital for cytotoxic T cell (CTL) activation [28]. This is of particular benefit when considering MVA as a vaccine vector for orthohantaviruses as a robust CTL response is thought to be critical in the clearance of orthohantavirus infections [29].

Orthohantavirus NPs have previously been shown to be strongly immunogenic, particularly against HTNV, demonstrating a role in long-term protection against HFRS causing orthohantaviruses [30,31]. NPs contain many of the epitopes needed to raise a strong CD8^+^ response and are highly conserved across orthohantaviruses and so induce a broad range of cross-reactivity, and ultimately protection, across a range of orthohantaviruses [32]. The nucleoprotein is also soluble and therefore easier to manipulate into a chimeric recombinant protein format, as soluble proteins tend to have more than one folding position, particularly compared to the GP structural proteins, which have one specific native assembly. This property will ensure the correct epitope formation for an effective and strong immune response. SEOV and HTNV are the main targets due to the global public health issues that these orthohantaviruses present.

This study therefore aimed to develop a suitable multi-valent hantavirus vaccine in an MVA vector and to perform pre-clinical immunogenicity and efficacy analysis in IFN-α/β-deficient mice.

## 2. Materials and Methods

### 2.1. Cells

CEF cells (Pirbright, UK) were cultured in Minimum Essential Media supplemented with 2% FBS, 2 mM L-glutamine, 100 U penicillin and 0.1 mg/mL streptomycin (Sigma, UK) at 37 °C 5% CO_2_. Vero E6 cells (European Collection of Cell Cultures, Salisbury, UK) were cultured in high-glucose Dulbecco’s modified eagle medium (DMEM, Sigma, Poole, UK) supplemented with 2% FBS (Sigma, UK) and incubated at 37 °C in 5% CO_2_.

### 2.2. Plasmids

**Figure 1:**
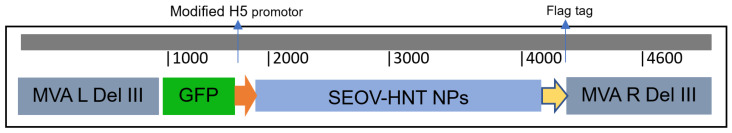
MVAHantaNP plasmid cassette.

pMVAHantaNP, produced by GeneArt (Thermofisher), is a pUC-based vector containing a GFP-tagged chimeric HNTV/SEOV NP (HantaNP) insert that was codon optimised for *Homo sapiens* for amino acids 37-1323 (HNTV) and 43-1332 (SEOV), respectively. To increase recombinant MVA construct stability, a consideration regarding the design of recombinant antigens was made to reduce any unnecessary sequence. Therefore, the N terminal part of the Hantaan N up to residue 94 was removed since this is represented by the sequence of SEOV N; although there are some amino acid differences between SEOV and Hantaan N in this region, the differences are very minor (i.e., closely related amino acids). The Hantaan N was started at residue 95 since this is a different residue from SEOV and ensures the inclusion of a length of Hantaan N to preserve what could be an important structural and antigenic feature of Hantaan N. While SEOV and Hantaan N proteins are generally very similar, a major difference occurs between the two at residues 241–300. The 3D structure of Hantaan N has been solved, showing that residues 241–300 are a section of polypeptide which is thought to fold in a specific fashion that connects two lobes of the N protein and starts the second lobe of the protein [33]. Because the amino acid sequence of the SEOV and Hantaan N in this region is different, we aimed to ensure that these differences were preserved in the recombinant antigen. Since this region may have some important structural context, we designed the Hantaan N to include the amino acids on either side to ensure as correct a folding framework as possible. The HantaNP insert also contained a p11 promoter, a GFP-tag and a modified H5 promoter followed by a Kozak sequence upstream of the chimeric HNTV/SEOV NP sequence, followed by a 23-residue linker sequence, a flag-tag epitope and a stop codon (Figure 1).

### 2.3. Viruses

MVA strain 1974 was kindly donated by Prof B Moss (US National Institutes of Health, Bethesda, MD, USA) and titrated by plaque assay on CEF cells, as previously described [34].

Seoul Humber Hantavirus was isolated from a rodent in Yorkshire in 2012 and titrated by TCID_50_ with RT-PCR readout, on Vero E6 cells as previously described [8,35]. Stocks were cultured in Vero E6 cells at 37 °C and 5% CO_2_ for 11 days in high-glucose DMEM supplemented with 2% FBS. Virus cultures were snap frozen at −80 °C and supernatant clarified by centrifugation at 3824× *g* for 10 min. Sanger sequencing and culture in Luria Bertani broth and Terrific Broth for 10 days at 37 °C were then performed to confirm that virus cultures were sterile and free from mycoplasma.

### 2.4. Vaccine Production

Recombinant MVA encoding the chimeric HantaNP insert (rMVAHantaNP) was produced in CEF cells. Briefly, sub-confluent CEF cells were infected with MVA 1974 at a multiplicity of infection (MOI) of 0.05. MVA-infected CEFs were then transfected with pHantaNP using Lipofectamine^®^ Transfection Reagent (Thermofisher, Dartmouth, UK) as directed by the manufacturer. Cell supernatant containing rMVAHantaNP was harvested 2 days post-transfection and snap frozen, followed by 3x freeze-thaw cycles prior to further processing. To obtain a homogeneous rMVAHantaNP population, four rounds of serial plaque-purification in CEF cells with a 2% agarose type V11 (Sigma, UK) overlay was carried out. This was followed by batch amplification in CEF cells and purification by sucrose cushion [36]. rMVAHantaNP was then titred by plaque assay on CEF cells incubated for 2 days with a 2% agarose type VII (Sigma, UK) overlay [37]. Viral foci were visualised by immunostaining with rabbit anti-vaccinia antibody (Ab Serotec, Kidlington, UK) and Vectastain Universal ABC-AP kit (Vector laboratories, Newark, CA, USA) as previously described [27].

### 2.5. Detection of Protein Expression

Sub-confluent CEF cells were infected with rMVAHantaNP at a MOI 0.05 and incubated at 37 °C with 5% CO_2_ in MEM supplemented with 2% FBS. After 48 h, when cells were close to confluence, GFP fluorescence was apparent, and CPE (cytopathic effect) was observed microscopically. The medium was aspirated, and cells were lysed with 1x LDS NuPAGE^®^ reducing sample buffer (Thermofisher, UK) and heated at 70 °C for 10 min. Lysates were then analysed via western blot on nitrocellulose membrane following gel electrophoresis on a NuPAGE^®^ 4–12% Bis-Tris gel (Thermofisher, UK). The membrane was blocked with 5% milk solution (Merck Millipore, Watford, UK) in phosphate-buffered saline (PBS) containing 0.05% Tween^®^20 (PBS-T, Sigma) and incubated with primary antibody (polyclonal rabbit anti-FLAG tag, Sigma) at 1:1000 in PBST-T for 1–2 h with rocking, before being washed three times in PBS-T. Membranes were incubated in the presence of the secondary antibody (anti-rabbit IgG HRP, Sigma) at 1:1000 in PBS-T for 1 h with rocking and washed as before. Protein expression was determined by the detection of bound antibody using a Pierce ECL WB substrate kit (Thermofisher, UK) according to the manufacturer’s instructions and visualised in a Chemi-Illuminescent Imager (Syngene, Cambridge, UK).

### 2.6. Animals

Adult female A129 (IFN-α/β R^−/−^) mice aged 6–8 weeks were supplied from an established breeding colony approved by the UK Home Office (Marshall Biosciences, East Yorkshire, UK). Food and water were available *ad libitum*, with environment enrichment included in cages. Animals were housed in the animal facility for over a week to acclimatise prior to any procedure being conducted.

All procedures with animals were undertaken according to the United Kingdom Animals (Scientific Procedures) Act 1986. These studies were approved by the ethical review process of the UK Health Security Agency (or its predecessors) and the Home Office, UK via an Establishment Licence (PEL PCD 70/1707) and project licence (P82D9CB4B).

### 2.7. Immunogenicity and Efficacy Studies

57 mice were divided into four groups through random allocation by animal facility staff blinded to the study: Group 1: 20 mice received a two-dose vaccination of rMVAHantaNP in endotoxin-free PBS at 1 × 10^7^ plaque-forming units (PFU) per dose on day 0 and day 14. Group 2: 20 mice received a single-dose vaccination of rMVAHantaNP in endotoxin-free PBS at 1 × 10^7^ PFU per dose on day 14. Group 3: 20 mice received a two-dose vaccination of MVA 1974 (wild-type) in endotoxin-free PBS at 1 × 10^7^ focus forming units (FFU) per dose on days 0 and 14. Group 4: 17 mice received a two-dose vaccination of endotoxin-free PBS only (negative control) on days 0 and 14.

All mice were injected intramuscularly (IM) into each caudal thigh; a total of 100 µL was administered at each vaccination (50 µL into each thigh). Animal weights and temperatures were recorded daily throughout the study.

On day 28, five animals from each group (two mice from Group 4) were euthanised, and samples of spleen tissue and blood were collected at necropsy for immunological assessment.

The remaining mice (*n* = 40) not culled on day 28 proceeded to form the efficacy study and so were challenged with SEOV. For each group, *n* = 5 animals were challenged via the intranasal (IN) route, and *n* = 5 animals were challenged via IM route at 1.36 × 10^6^ TCID^50^/dose. Challenge was given under isoflurane sedation, and animals monitored until a full recovery from sedation was observed. On day 33 (5 days post-challenge), animals were culled, and blood, liver, kidney, spleen and lung tissues collected for viral burden analysis.

### 2.8. Interferon-Gamma ELISPOT

Spleens from mice culled on day 28 of the immunogenicity study were collected aseptically and homogenised, and red blood cells lysed. Splenocytes were then resuspended in RPMI medium (Sigma, UK) supplemented with 5% FBS, 2 mM L-Glutamine, 100 U penicillin & 0.1 mg/mL streptomycin, 50 mM 2-mercaptoethanol and 25 mM HEPES solution (Sigma, UK). Splenocytes were assessed for their response when exposed to HTNV/SEOV antigens via IFN-γ ELISpot (Mabtech, Nacka Strand, Sweden), performed as per the manufacturer’s instructions. Briefly, splenocytes were seeded in PVDF microtiter plates at 1.86 × 10^5^ cells per well and re-stimulated with peptide pools (JPT, Berlin, Germany). Peptide pools contained 16–18 15mer peptides, each at 2.5 µg/mL, and provided coverage across the HTNV/SEOV vaccine antigen, with a 10 residue overlap between each peptide.

Plates were developed after 18 h at 37 °C, 5% CO_2_ as per manufacturer’s instructions, with IFN-γ response quantified on an automated ELISpot reader (Cellular Technologies Limited, Cleveland, OH, USA). Values acquired from control wells (containing splenocytes and medium only) were subtracted from counts acquired from wells stimulated with peptides, and results are expressed as spot forming units (SFU) per 10^6^ cells.

### 2.9. Antibody ELISA

Immulon 2HB 96-well plates (Thermofisher, UK) were coated overnight at 2–8 °C with 200 ng/well GST-tagged HTN-SEOV-NP protein (Oxford Expression Technologies, Oxford, UK) in 0.2 M carbonate-bicarbonate buffer, pH 9.4 (Thermo Scientific, Loughborough, UK). Plates were then washed with PBS + 0.01%Tween^®^20 (PBS-T) (Sigma, UK) and blocked in 5% milk powder (Merck, Millipore, UK) diluted in PBS-T for 1 h at 37 °C, before re-washing in PBS-T. Serum samples were diluted in PBS-T supplemented with 5% (*w*/*v*) milk powder, added at a 1:100 dilution with 100 µL/well and incubated at 37 °C for 60 min. Plates were washed as before and probed with 100 µL/well of HRP-conjugated donkey anti-mouse IgG antibody (Sigma, UK) diluted to 1:20,000 in 5% milk powder in PBS-T. Plates were incubated for 60 min at 37 °C, washed with PBS-T and binding visualised using TMB substrate and stop solution as per manufacturer’s instructions (Surmodics, Eden Prairie, MN, USA). Plates were read at 450 nm using an absorbance reader and analysed with the Softmax Pro version 5.2 software (Molecular Devices, San Jose, CA, USA). Any OD value greater than the mean value plus three Standard Deviations (SD) of the data acquired from the PBS only control group (Group 4) were considered positive.

### 2.10. Sample Processing

Samples and tissues that were collected from mice were immediately frozen at −80 °C prior to viral load analysis. Blood was collected into RNAprotect tubes (Qiagen, Manchester, UK), and saliva into dry tubes. Tissue samples were placed into tubes containing RNAlater (Qiagen, UK), weighed and homogenised using ceramic beads and an automated PreCellys-24 homogeniser (Bertin Scientific, Basingstoke, UK); they were then inactivated by the addition of RLT (Qiagen, UK) containing β-mercaptoethanol (Sigma, UK) followed by 70% ethanol (Sigma, UK) and fumigation. Once inactivated, tissue samples and biological fluids were further homogenised via QIAshredder (Qiagen, UK) and extracted using the BioSprint 96 One-For-All Vet extraction kit (Qiagen, UK).

### 2.11. qRT-PCR Assay

A qRT-PCR assay specific to the SEOV and HTNV genomes was used to detect viral RNA. The assay used is based on the method published by Kramski et al. [37], using HNTV/SEOV F and HTNV/SEOV R primers and a variation of the probe HNTV/SEOV TMGB2 (nucleotide 9, A, was replaced with degenerative purine nucleotide, R (see bold text in sequence)), to give HNTV/SEOV TMGBX: FAM-TCAATGGG**R**ATACAACT-NFQ-MGB (TMGB refers to hydrolysis probe coupled to a Minor Groove Binder (MGB) moiety and NFQ, the non-fluorescent quencher at 3′ end).

RT-PCR reactions were carried out in a 20 µL reaction volume containing 900 nM HTNV/SEOV F, 450 nM HTNV/SEOV R, 375 nM HTNV/SEOV TMBX, 5.5 mM MgSO_4_, 1X Reaction Mix, 0.8 µL SuperScript^TM^ III RT/Platinum^TM^ *Taq* Mix and 5 µL template. RT-PCR cycling was carried out on the ABI 7500 platform using the following parameters: 50 °C for 10 min, followed by 95 °C for 2 min and 45 cycles of 95 °C for 10 s and 60 °C for 40 s. Analysis was carried out using the QuantStudio™ Real-time PCR software (version 1.6.1) and auto-baseline setting, with the ΔRn threshold set to 200,000. RNA levels were quantified against a standard curve. Samples showing exponential amplification outside the linear range of the assay (<1 × 10^1^ copies/µL) were classified as hantavirus RNA detected at a low level and assigned the value 25 to enable statistical analysis.

Viral burden was expressed as genome copies per gram (g) or per millilitre (mL).

### 2.12. Statistical Analysis

To determine statistical significance between groups, two-way ANOVAs were performed, and, for intra-group comparison, Mann–Whitney analyses were performed on data. These analyses were carried out on GraphPad Prism software (Version 7.0, GraphPad software, Bishop’s Stortford, Hertfordshire, UK). Differences were considered to be statistically significant if *p* ≤ 0.05.

## 3. Results

### 3.1. Production of Vaccine Candidate

**Figure 2:**
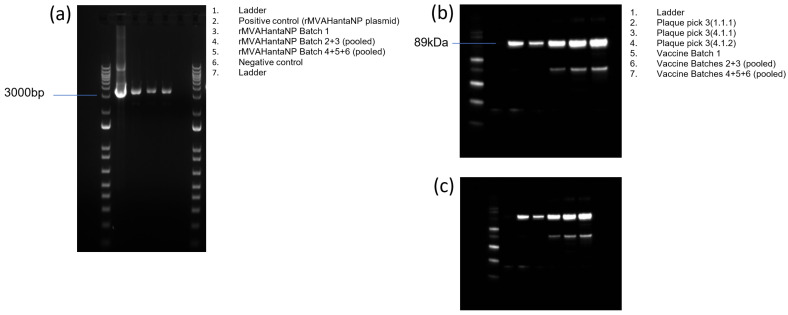
Confirmation of antigen incorporation into MVA. (**a**) PCR using primers designed specifically to confirm the presence of the Hantavirus nucleoprotein to the MVA flanking region with an expected size of 3260 bp in each of the vaccine batches. (**b**) Western blot with anti-flag staining antibodies for the detection of antigenic insert. (**c**) Western blot after staining with antibodies against Hantavirus nucleoprotein.

A recombinant MVA-based vaccine encoding HTNV and SEOV NP (including a FLAG-tag and GFP) as a chimeric vaccine antigen was produced in CEF cells. End-point PCR with primers targeting the MVA flanking regions confirmed the presence of pure recombinant MVA containing HantaNP insert, with a clear band visible at 3260 bp. There was also no evidence of reversion to MVA-WT as a band at 549 bp was not visible (Figure 2a). The expression of the inserted vaccine antigen (HantaNP) was confirmed by western blot, with a clear band at approximately 90 kDa, corresponding to the 89 kDa FLAG-tagged HantaNP insert (Figure 2b). There is also a smaller, fainter band at approximately 50 kDa that can be seen on the western blot in lanes 3, 5, 6 and 7; this band was also seen when samples were labelled using the anti-HantaNP antibody that recognises both HTNV and SEOV NP epitopes (Figure 2c). This gives us confidence that this band is likely to be a truncated version of the insert.

**Figure 3:**
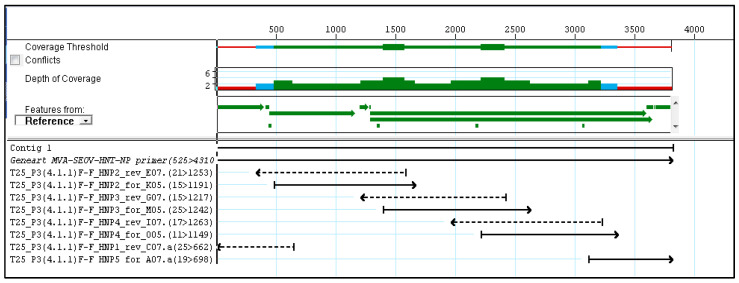
Sequence analysis of plaque pick 3 (4.1.1) demonstrating sufficient coverage compared to reference sequence with no additions of stop codon or major mutations.

Sequencing analysis of Plaque pick 3 (4.1.1) demonstrated coverage across the majority of the sequence (Figure 3). The MVA flanking regions are not covered. The overall sequence matches the original with no additions of stop codon or any mutations. This suggests that the insert is suitable for vaccine production.

### 3.2. Immunogencity

#### 3.2.1. Vaccine Tolerability

Over the 42-day study period, the vaccine was well tolerated, with all mice showing no clinical signs of disease and scoring healthy throughout.

#### 3.2.2. Humoral Immunity

**Figure 4:**
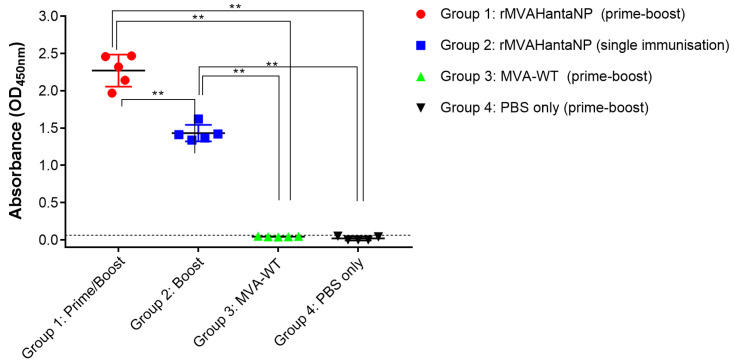
Hantavirus nucleoprotein-specific IgG levels detected via indirect ELISA in the serum of rMVAHantaNP-immunised mice compared to controls. Dotted line indicates cut-off level (mean value from control group ± 3 standard deviations). **, *p* < 0.01.

Serum collected 14 days post-vaccination was assessed to determine the humoral response to vaccination. Samples were analysed by ELISA for the presence of HantaNP-specific IgG antibodies. A positive result was deemed as an optical density (OD) greater than the mean plus 3SD of control group 4 values acquired (0.0629 OD^450^). Using these criteria, mice vaccinated with MVA-WT (control group 3) were not positive for anti-HantaNP IgG antibodies. There was also no significant difference between the two control groups (*p* > 0.05, Mann-Whitney analysis) (Figure 4). Serum from all mice in both groups vaccinated with rMVAHantaNP (groups 1 and 2) were positive for antibodies specific to Hanta-NP. Values acquired from group 1 were significantly higher compared to those acquired from the single-dose regime (*p* < 0.05, Mann–Whitney analysis).

#### 3.2.3. Cellular Immunity

**Figure 5:**
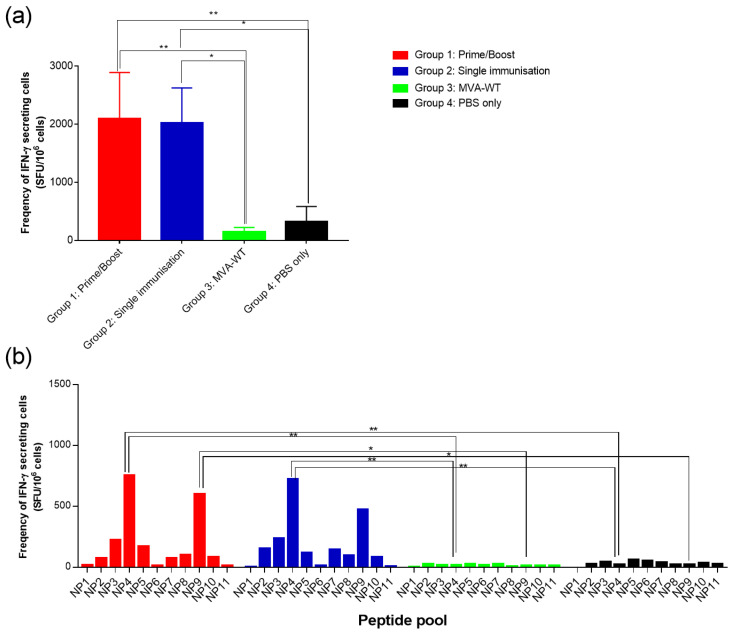
IFN-γ ELISPOT responses from A129 mice immunised with rMVAHantaNP after stimulation with peptides. (**a**) Summed responses from each vaccine group, showing mean values with error bars denoting standard error. (**b**) Responses from each group to each of the individual peptide pools. * *p* < 0.05, ** *p* < 0.01.

Splenocytes taken 14 days post-vaccination were analysed for the presence of HTNV/SEOV NP-induced IFN-γ by ELISpot to assess cell mediated immunity (CMI). When peptide responses were summed for all pools, rMVAHantaNP-vaccinated groups expressed statistically significant (*p* < 0.05, Mann–Whitney analysis) higher numbers of antigen-specific IFN-γ-secreting cells than the control groups (groups 3 and 4), with a mean of 191.72 SFU/10^6^ cells for group 1 and 183.64 SFU/10^6^ cells for group 2 vs. a mean of 13.75 SFU/10^6^ cells for group 3 and 29.1 SFU/10^6^ cells for group 4 (Figure 5a). Interestingly, there was no statistical difference between frequencies of IFN-γ-secreting cells in group 1 (prime-boost dosing schedule) and group 2 (single dose only) (*p* > 0.999, Mann–Whitney analysis) (Figure 5a).

When individual peptide pools were analysed, the distribution of IFN-γ response in rMVAHantaNP-vaccinated mice (groups 1 and 2) were significantly higher (*p* < 0.05, two-way ANOVA) following stimulation with peptide pools NP4 and NP9, when compared to control groups and responses to other peptide pools (Figure 5b). Control groups vaccinated with MVA-WT (group 3) and PBS (group 4) showed no notable response to any peptide pools.

### 3.3. Efficacy

**Figure 6:**
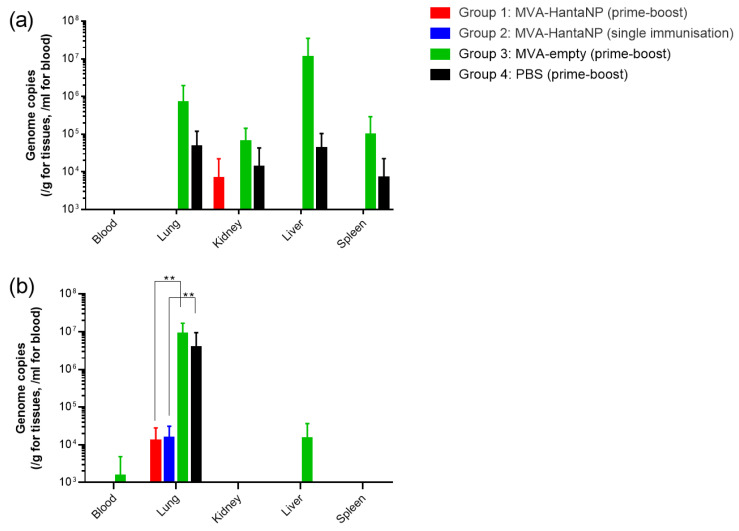
Viral RNA levels in blood and tissues collected from immunised mice 5 days post-challenge with SEOV via the (**a**) intramuscular and (**b**) intranasal route. Bars show mean values with error bars denoting standard error. ** *p* < 0.01.

As A129 mice are a non-fatal model for orthohantaviruses, efficacy was measured by reduction of viral burden. At 5 days post-SEOV challenge, animals were culled from each group to enable viral load and virus dissemination to be assessed. After IM challenge, viral RNA was detected across multiple tissues of mice in control groups (Group 3 and 4) (Figure 6a). In group 1, which received a prime-boost vaccination with rMVAHantaNP, only 1/5 mice challenged with SEOV had detectable SEOV RNA, and this was present only in the kidney, at 3.4 × 10^4^ copies/g. In group 2, no mice had detectable levels of SEOV RNA in any of the tissues or serum samples tested (Figure 6a).

In contrast, at 5 days post-IN challenge, mice in all 4 groups showed viral burden in the lung tissue (Figure 6b). This burden was most notable in the control groups (groups 3 and 4), with significantly less viral RNA in the rMVA-HantaNP-vaccinated groups (groups 1 and 2). Mice in group 3 also exhibited viral RNA in the liver (n = 2/5, and blood (1/5) (Figure 6b).

## 4. Discussion

This study has demonstrated that rMVAHantaNP may be a suitable vaccine candidate to reduce the severity of Seoul virus infections. As well as safety and efficacy, this vaccine was designed and constructed with ease of production, on a large scale, with minimal cost to patient in mind. With this specification, NP was an excellent vaccine antigen candidate: NPs have been shown to have a role in protection via the development of a robust CMI response against hantaviruses [30,31].

Western blot analysis demonstrated that the rMVAHantaNP was able to express the recombinant NP protein over multiple passages, demonstrating its stability and suitability as a vaccine candidate. Sequencing data demonstrated that the MVA vector containing HantaNP insert remained stable over multiple passages. Sequencing and RT-PCR data also demonstrated that the vaccine batch used in this study was pure, with no wild-type MVA present.

The MVA-vectored vaccine was well tolerated by mice which remained healthy after immunisation. Mice receiving rMVAHantaNP vaccine as a single dose and in a prime-boost approach were positive for anti-HantaNP IgG, which were statistically significantly higher responses compared to control groups receiving wildtype MVA or PBS control. The prime-boost regime demonstrated significantly increased values of HantaNP-specific antibodies compared to the single-dose regime. It is not known whether a humoral immune response is important for combatting SEOV or HNTV infection, but elevated IgG has been seen previously in other hantavirus infections and used as an indicator for disease severity in Puumala infections: increased levels of IgG suggest a milder form, whereas lower levels of IgG often suggest a more severe disease presentation [38]. Neutralising antibodies have been shown to be important for reducing the longevity of disease in Sin Nombre virus and have also been detected in survivors of Andes virus and Sin Nombre virus [39]. These findings suggest that IgG has a role in reducing the severity of infection, although their target antigens are likely to be GP, especially for neutralisation activity and the role of NP-specific antibodies is less clear.

As well as a humoral response, a cell-mediated response was seen when splenocytes processed from immunised animals were re-stimulated with HantaNP-specific antigens. CMI is thought to confer protective immunity against Hantaviruses [29], and NP has previously been shown to be the most relevant epitope for CTL recruitment in hantavirus infection [40]. Mice immunised with the rMVAHantaNP vaccine showed statistically significant higher frequencies of antigen-specific IFN-γ-secreting cells when compared to non-immunised animals, but unlike with the humoral response there were no significant differences between the single-immunisation and prime-boost approaches. There were two peptide pools in particular that the immunised groups gave significant responses when compared with other peptide pools: NP4 and NP9. NP4 codes for peptides within the Seoul region and NP9, the Hantaan region of the HantaNP chimeric protein; this shows that the vaccine has successfully raised a cellular immune response to both NP proteins and further confirms that both NP proteins within the chimeric protein sequence are being correctly expressed and presented to the immune system. It can be concluded that rMVAHantaNP raises a CMI to both hantavirus strains, Seoul and Hantaan virus, after a double or single immunisation of rMVAHantaNP.

Rodents are a natural host for Orthohantaviruses and do not show any clinical symptoms when exposed to hantavirus infection. This can make assessment of vaccine candidate studies challenging. However, we have previously shown that A129 mice do appear to accumulate virus in certain tissues when challenged with SEOV, particularly noticeable when infected via the intranasal route [34]. Tissue taken from mice culled on day 5 post-challenge were therefore assessed for viral load as a marker of protection against infection. After intramuscular challenge, only a single rMVAHantaNP mouse in the prime-boost group showed viral burden of any kind (in kidney tissue). This suggests that the vaccine effectively cleared residual SEOV, even on a single-dose schedule, when challenged intramuscularly, which would be representative of viral transmission via a bite or scratch, which, although relatively uncommon, has been reported [11]. After IN challenge, there was some viral RNA detected in immunized mice in the lung tissue from 3/5 mice per group with a viral burden range of 8.93 × 10^4^–3.64 × 10^4^ copies/g and 1.79 × 10^4^–3.6 × 10^4^ copies/g for those immunised in a prime-boost or single approach, respectively. In unvaccinated groups a more widespread burden was observed, with viral load detected in the blood and liver as well as the lungs (viral burden across lung tissue: 2.23 × 10^6^–1.82 × 10^6^ copies/g in the wild-type MVA group and 4.03 × 10^6^–1.25 × 10^7^ copies/g in the PBS control group. Therefore, vaccinated groups demonstrated a minimum of a two-log difference reduction in viral burden when compared to unvaccinated groups.

These results suggest that a single-dose vaccine stimulates an effective immune response that is sufficient to reduce viral burden from IN and IM challenge, which is more representative of the natural transmission of the virus from rodents to rodents (and humans). This correlates with the data acquired from stimulating splenocytes with NP peptide pools post-immunisation and measuring IFN-γ response, where there was no statistically significant difference between those animals receiving one dose of vaccine versus those in a prime-boost approach, implying that CMI is more critical in the clearance of SEOV in mice than the humoral immune response, as alluded to by the ELISA data which demonstrated statistically significantly different levels of HantaNP-specific antibodies between the two immunised groups.

Although the mice were challenged with SEOV only, with the observation of an HTNV cellular immune response, it can be extrapolated that the vaccine also protects against HTNV. In future studies it would be pertinent to confirm this with an HTNV challenge. Cross protection has been seen within Hantavirus families from the same region (Old World or New World Hantaviruses) [29], and, as previously mentioned, helper T-cell epitopes in HFRS survivors are highly conserved across hantaviruses [40]. Cellular immunity via activation of cytotoxic t-cells is thought to be imperative for clearance of hantavirus infection in humans [16].

The market for the prevention and treatment of HFRS is very limited: Hantavax™, although not licensed in Europe, is the only vaccine currently available against hantavirus globally. It is also specific to HFRS-causing Hantaan and Seoul viruses and requires three doses. Hantavax™ has been shown to have a reduced immunogenicity after 735 days (according to PRNT^50^); in addition, there are safety concerns including serious adverse events, and it is now only recommended for people at high risk of exposure [20].

Ribivirin is currently the only treatment option available in Europe and, if administered within 5 days of infection, can reduce the likelihood of patient fatality, but even with increasing awareness regarding hantaviruses across Europe, diagnosis is not always as timely as this, and so, once the diagnosis has been made, the 5-day deadline is likely to have passed. Ribivirin is expensive and also has side effects such as anaemia when used therapeutically. Having said that, if an outbreak were to occur, Ribivirin is currently our only available choice, so another, more viable option is required.

The rMVAHantaNP vaccine has demonstrated its ability to raise both a humoral and a CMI response. The vaccine was able to reduce viral load after a single-dose immunisation in tissues compared to unvaccinated mice. This suggests that the vaccine stimulates the appropriate immune response to reduce the presence of hantavirus infection in mice. All mice tolerated the vaccine well and remained healthy throughout the study. The data from this research have further confirmed MVA as a suitable, safe and effective vaccine vector.

## 5. Conclusions

The research carried out demonstrates that the rMVAHantaNP vaccine candidate has the potential to become an effective vaccine against SEOV and HTNV infection. The data show that the vaccine stimulates both a humoral and a cell-mediated immune response, both of which were effective in reducing SEOV burden in tissues post-challenge. A larger cohort study and ideally a lethal animal model of HFRS disease needs to be established to fully demonstrate efficacy and protection, to further support pre-clinical and clinical trial development.

## Data Availability

The original contributions presented in the study are included in the article, further inquiries can be directed to the corresponding author.
